# Minimal Disease Monitoring in Pediatric Non-Hodgkin’s Lymphoma: Current Clinical Application and Future Challenges

**DOI:** 10.3390/cancers13081907

**Published:** 2021-04-15

**Authors:** Lara Mussolin, Christine Damm-Welk, Marta Pillon, Wilhelm Woessmann

**Affiliations:** 1 Department of Women’s and Children’s Health, Clinic of Pediatric Hemato-Oncology, University of Padova, 35128 Padova, Italy; lara.mussolin@unipd.it (L.M.); marta.pillon@unipd.it (M.P.); 2 Istituto di Ricerca Pediatrica, Fondazione Cittàdella Speranza, 35127 Padova, Italy; 3 Pediatric Hematology and Oncology, University Medical Center Hamburg-Eppendorf, 20246 Hamburg, Germany; c.damm-welk@uke.de

**Keywords:** non-Hodgkin lymphoma, children and adolescents, minimal disseminated disease, minimal residual disease, clinical application

## Abstract

**Simple Summary:**

Minimal residual disease (MRD) describes the detection of a few remaining malignant cells in blood or bone marrow with molecular methods or flow cytometry. The detection of MRD in patients with leukemia during therapy indicates the risk of relapse. Its use for treatment stratification is state of the art in all modern leukemia treatment protocols. In pediatric non-Hodgkin lymphoma patients, minimal amounts of tumor cells, so-called minimal disseminated disease (MDD), can often be detected in blood or bone marrow at diagnosis. In children with ALK-positive anaplastic large cell lymphoma, MDD and MRD detected in the blood or bone marrow is associated with high relapse risk. For patients with Burkitt lymphoma or -leukemia or lymphoblastic lymphoma, the meaning of MDD and MRD is less clear. This review summarizes the current knowledge, techniques, application, and challenges in minimal disease detection in pediatric non-Hodgkin lymphoma.

**Abstract:**

Minimal residual disease (MRD) detection is established routine practice for treatment stratification in leukemia and used for treatment optimization in adult lymphomas. Minimal disease studies in childhood non-Hodgkin lymphomas are challenged by stratified treatment in different subtypes, high cure rates, low patient numbers, limited initial tumor material, and early progression. Current clinical applications differ between the subtypes. A prognostic value of minimal disseminated disease (MDD) could not yet be clearly established for lymphoblastic lymphoma using flow cytometry and PCR-based methods for T-cell receptor (TCR) or immunoglobulin (IG) rearrangements. *MYC–IGH* fusion sequences or IG rearrangements enable minimal disease detection in Burkitt lymphoma and -leukemia. An additional prognostic value of MDD in Burkitt lymphoma and early MRD in Burkitt leukemia is implicated by single studies with risk-adapted therapy. MDD and MRD determined by PCR for ALK-fusion transcripts are independent prognostic parameters for patients with ALK-positive anaplastic large cell lymphoma (ALCL). They are introduced in routine clinical practice and used for patient stratification in clinical studies. Early MRD might serve as an endpoint for clinical trials and for guiding individual therapy. Validation of MDD and MRD as prognostic parameters is required for all subtypes but ALCL. Next-generation sequencing-based methods may provide new options and applications for minimal disease evaluation in childhood lymphomas.

## 1. Introduction

Non-Hodgkin lymphomas (NHLs) account for 8% of childhood and adolescent malignancies and comprise a heterogeneous group of different diseases. The major subtypes in children and adolescents are the mature B-cell lymphomas Burkitt lymphoma/leukemia (BL, B-AL, 45%) and diffuse large B-cell lymphomas (DLBCL, 10%), lymphoblastic lymphomas (LBL, 20%) and ALK-positive anaplastic large cell lymphomas (ALCL, 15%) [1]. Rarer entities observed in more than 1% of patients are primary mediastinal B-cell lymphomas (PMBCL), peripheral T-cell lymphomas (PTCL), pediatric type follicular lymphomas, and pediatric nodal marginal zone lymphomas. Therapeutically, three subgroups are usually distinguished: mature B-NHL (Burkitt and DLBCL), LBL, and ALCL. With current subtype-specific treatment strategies, the event-free survival (EFS) rates at five years reach 90% for mature B-NHL, 80% for LBL, and 70% for ALCL [2,3,4,5]. Relapses usually occur very early during or after the end of initial therapy. In BL/B-AL and LBL, relapses often present as refractory disease, with survival rates in relapse of only 20%, whereas patients with relapsed ALCL or DLBCL have a fair chance to survive [6,7,8,9].

Minimal residual disease (MRD), the detection and quantification of submicroscopic systemic disease during or after therapy, has been established as the most powerful prognostic factor and tool for disease monitoring in childhood acute lymphoblastic leukemia (ALL). Stratification according to MRD belongs to standard clinical practice [10,11,12]. During recent years, minimal disseminated disease (MDD) and MRD have also been extensively investigated in adult DLBCL [13,14].

The revised international pediatric non-Hodgkin lymphoma staging system (IPNHLSS) proposes to also collect additional information on minimal disease for children with NHL in order to allow the integration of advanced and more sensitive technologies in the analysis of bone marrow (BM), blood (PB), or central nervous system involvement in the future [15]. However, several challenges are faced when studying minimal disease in children with NHL. In contrast to ALL, for which both flow cytometric and molecular MRD methods are established with international quality control, MRD methods have to be adapted for each biological subgroup necessitating the development and validation of different methods for limited patient populations. Abundant initial tumor material to establish MRD markers is available for leukemia. In contrast, this material often is scarce in patients with lymphoma and needs to be reserved for assurance of diagnosis. Fresh tumor material can rarely be collected for marker screening from lymphoma biopsies. Except for bone marrow involvement in BL, LBL, and PTCL, as well as B-AL, initial systemic disease detection cannot be taken as given. In most instances, screening for minimal disseminated disease (MDD), defined as submicroscopic detection of tumor cells in blood or bone marrow, is required as a prerequisite for studying MRD during therapy or for relapse monitoring. MDD itself needs to be investigated for its possible prognostic value for each lymphoma subtype comparable to micrometastases in solid tumors or adult DLBCL [16,17,18,19,20].

Further specific characteristics have to be considered when studying MDD and MRD as risk factors for treatment stratification in childhood NHL. The high cure rate in most NHL subtypes necessitates prognostic parameters with very high predictive value in order to enable patient stratification. In addition, MRD needs to be studied very early during the course of initial treatment, given the time of relapse/progression in childhood NHL.

Here, we summarize the techniques currently used for minimal disease detection, the available clinical data on MDD and MRD’s prognostic meaning for the major NHL subtypes in children and adolescents, and discuss new developments and applications of MDD and MRD.

## 2. Current Status of MDD and MRD in Lymphoblastic Lymphoma (LBL)

The majority of LBL arises from immature T cells [21], 20% are of B-precursor cell origin. The antigen binding domains of T-cell receptors (TCRs) and immunoglobulins (IGs) are unique for each lymphocyte and serve as patient-specific clonal markers. Initial fresh lymphoma material is needed to screen for these rearrangements in each case with the identification of clone-specific junctional sequences. Junctional region-specific oligonucleotides need to be designed, which are used as primers for the PCR assay for MDD/MRD evaluation with a sensitivity of 10^−5^. To bypass this problem, MDD could be investigated by flow cytometry (FCM) since it usually does not require initial fresh tumor material [22,23]. LBL cells are recognized for co-expression of cell markers not found in normal lymphocytes or typical of lymphocytes normally confined to the thymus. Stark et al. reported a good correlation between PCR-based and FCM-based methods in T-LBL [24]. The first data on the prognostic impact of MDD in T-LBL, evaluated by FCM, was reported by Coustan-Smith et al. in 99 pediatric patients [22]. MDD was detected in 72% of BM studied (71/99), with T-lymphoblasts ranging from 0.01% to 32%. Fifty-five of the 71 T-LBL-positive samples were from patients with St. Jude stage II/III disease. Detection of MDD in BM was more frequent among younger patients (<10 years; *p* = 0.046) and among patients with lower lactate dehydrogenase (*p* < 0.01). Comparable to T-ALL, MDD in blood and BM correlated. Using a cut-off level of 1%, the 2-year EFS of 26 patients with higher levels of disease dissemination was 68% versus 91% for those with lower levels. The 2-year EFS of patients with detectable MDD up to 0.1% did not differ from the EFS of patients with negative MDD. In a multivariate analysis including all 99 patients, detection of T-LBL cells by FCM did not retain an independent prognostic value.

The Italian Association of Pediatric Hematology-Oncology (AIEOP) study group examined initial BM and peripheral blood (PB) samples from a series of 65 children affected by T- (52) and B-lineage (13) LBL using FCM [23]. MDD was detected in 49% (32/65) of BM samples. Paired BM and PB samples also demonstrated concordant results in this study. Using an MDD cut-off level of 3% (75th percentile), 5-year EFS was 60 ± 22% for patients with MDD > 3% versus 83 ± 6% for the remaining patients (*p* = 0.04), whereas other clinical parameters were without prognostic information.

These results cumulatively indicate that MDD assessment in LBL by FCM with a sensitivity of 10^−4^ can be based on blood. Since FCM can be performed faster and is less expensive compared to a quantitative real-time PCR (RQ-PCR) assay for TCR or IG rearrangements and does not necessarily need initial tumor tissue, FCM-MDD can be applied to most LBL patients.

Whether RQ-PCR-based quantification of MRD in MDD-positive LBL patients could enable detection of very high-risk patients amenable for therapy intensification, like in ALL, has not been studied so far.

The current European LBL 2018 study stratifies children with T-LBL according to *FBXW7* and *NOTCH-1* mutational status, which is determined from FFPE material [25,26,27]. MDD detection based on these tumor- and patient-specific molecular markers could be tested for the 60% of T-LBL patients with a mutation. Studying whether MDD or MRD could provide an additional prognostic value among the low-risk patients with mutated *FBXW7* or *NOTCH-1* needs, however, large patient numbers.

MRD, measured by either FCM or PCR-based techniques, could also be used to overcome the difficulty in morphologically distinguishing malignant lymphoblasts from non-malignant regenerating cells (hematogones) in BM during chemotherapy for LBL.

## 3. Current Status of MDD and MRD in Burkitt Lymphoma/Leukemia (BL/B-AL)

BM is involved in 25% of patients with BL, 80% of whom have a B-AL defined by more than 25% L3 blasts in the BM. The high proliferation rate of BL of virtually 100% is associated with a very high apoptotic rate prohibiting MDD/MRD analysis by FCM after shipment to a central reference laboratory.

Genetically, BL and B-AL are characterized by the presence of chromosomal translocations involving the *C-MYC* gene on chromosome 8 and the immunoglobulin heavy or light chain genes on chromosome 14, 22, or 2 [28]. The most common translocation, accounting for almost 80% of all cases, is the t(8;14)(q24;q32), which juxtaposes the *MYC* gene to the immunoglobulin heavy chain (*IGH@*) locus on chromosome 14 in divergent orientation. The *MYC-IGH@* rearrangement leads to the overexpression of C-MYC and is detectable at the genomic DNA level. In the majority of sporadic BL, the *MYC-IGH@* fusion can be detected by a long-distance polymerase chain reaction (LD-PCR) assay, which relies on the use of one primer specific for *C-MYC* exon 2 combined, in different reactions, with four primers for the *IGH@* locus [29]. The LD-PCR assay is useful not only for the molecular characterization of the primary disease but can also be applied to detect MDD/MRD because the breakpoint is specific for each individual tumor and patient. The assay has a sensitivity approaching 10^−3^ to10^−4^ both in vitro and in vivo [29]. Notably, it is not possible to study endemic BL by this technique due to the large chromosomal region involved. The AIEOP study group used an LD-PCR-based assay for the *MYC–IGH* fusion to prospectively study a cohort of 134 BL specimens [30]. This large cohort confirmed earlier data that 65–70% of BL have a detectable *MYC-IGH* fusion by the LD-PCR and that 30% of patients with available BM were MDD-positive, whereas only half of them were positive by cytology. Most of the patients with molecular detection of disease in the BM at diagnosis (22/26, 85%) belonged to the R4 risk group according to the Berlin-Frankfurt-Muenster (BFM) definition (stage III or stage IV according to St. Jude staging classification and LDH ≥ 1000 U/L). The 3-year progression-free survival (PFS) was 68 ± 10% for MDD-positive R4 patients compared with 93 ± 5% for MDD-negative R4 patients (*p* = 0.03) [30], whereas there was no significant difference in PFS between children with morphological BM involvement at diagnosis and those without. By multivariate analysis, only MDD was predictive of a higher risk of failure among R4 patients (hazard ratio, 4.7; *p* = 0.04). These data could be validated in a prognostic factor study of the AIEOP, including 128 BL patients with available MDD analysis in BM treated with the AIEOP LNH-97 protocol [31].

In one smaller study, MDD correlated with BM and PB samples, suggesting the possibility of monitoring MDD by blood testing in the future [32].

The application of the LD-PCR assay has the limitation to be applicable to about 80% of patients with t(8;14) but not to patients with t(2;8) or t(8;22), so that altogether, about 2/3 of patients with BL can be analyzed. This obstacle can be overcome at least in part using clone-specific IG gene rearrangements as MDD target [33]. Initial tumor material needs to be screened for these rearrangements in each patient. These assays were performed in 36 B-AL and 19 BL cases by the AIEOP [34]. In 88% of the cases, an RQ-PCR assay with a sensitivity of at least 10^-4^ could be established. Molecular BM involvement at diagnosis was detected in one-third of BLs using this assay comparable to the results with the tumor-specific long-distance PCR. MRD positivity persisted during chemotherapy in 6/36 children affected by B-AL. In most patients, LD-PCR and *IG* gene rearrangement-based methods detected MRD with similar results [33]. Thus, both methods can be used for MDD/MRD analysis in mature B-AL and BL patients with inherent advantages and disadvantages. The LD-PCR method is fast and relatively inexpensive but can be used for about 70% of patients, has limited sensitivity, and is reported semi-quantitatively. IG rearrangements are near-universal targets for MRD studies in B-cell malignancies and provide accurate quantification of MRD, but their detection is laborious. Both methods require initial fresh tumor material for target detection or validation, which is not available for a large part of patients with BL. To overcome this limitation, the feasibility of using IGV(H) primer pools to detect disease in clinical specimens was assessed. IGV(H) primer pools from IGV(H1)–IGV(H7) regions were tested to detect MDD/MRD, thus eliminating the need for an original tumor. Until now, only small cohorts of patients have been analyzed [35,36]. Agsalda et al. hypothesized that MRD could be screened in specimens using primer pools made up of IGV_H_ oligomers from respective V_H1_ to V_H7_ families [35]. The study was limited to 14 patients, but the findings support the feasibility of this approach because a previous study using patient-specific primers on the same cohort of children gave concordant results. Overall, MDD/MRD detection by IGV(H) primer pools needs further investigation.

**Figure 1:**
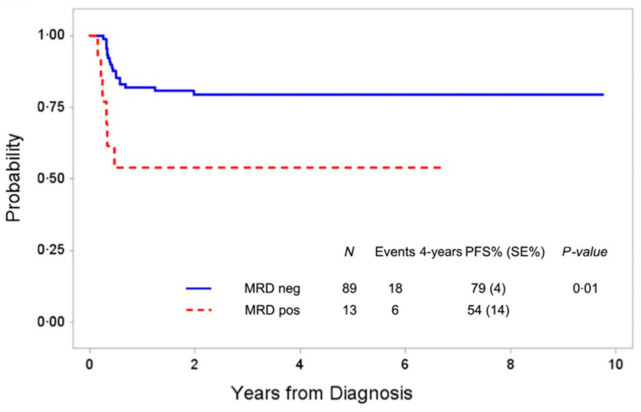
Four-year progression-free survival (PFS) of 102 children with Burkitt leukemia or stage IV Burkitt lymphoma according to minimal residual disease before the second course of Berlin-Frankfurt-Muenster (BFM)-type chemotherapy as measured by long-distance PCR for *MYC-IGH* or patient-specific IG rearrangements (from Mussolin et al. [38]).

There is very limited information on the prognostic relevance of MRD in B-AL, possibly due to its rarity. The AIEOP group detected the presence of t(8;14) by LD-PCR in 69% of 68 BM at diagnosis from children with B-AL [37]. MRD response before the second course of chemotherapy was determined in 39 patients. The 3-year relapse-free survival was 38 ± 7% for patients who were MRD-positive after the first chemotherapy cycle compared with 84 ± 7% for MRD-negative patients (*p* = 0.0005). The negative prognostic impact of early MRD persistence could be confirmed by the same group in 102 patients with B-AL or stage IV BL using either the LD-PCR or IG rearrangement for MRD detection, as shown in Figure 1 [38].

Given the efficacy of front-line therapy and the poor survival at relapse, very strong prognostic parameters are needed to detect the patients with a high relapse risk available for early clinical studies with experimental drugs. MDD and MRD might serve as tools to identify patients at risk if the encouraging data from AIEOP can be validated in an independent cohort of patients. Both the LD-PCR for *MYC-IGH* and the IG rearrangements have the abovementioned advantages and disadvantages, so further refinement of quantification and the use of initial FFPE material for marker screening are short-term goals for MRD development in BL and B-AL.

## 4. Current Status of MDD and MRD in Anaplastic Large Cell Lymphoma (ALCL)

ALK-positive ALCL in children and adolescents is characterized by translocations involving the ALK gene on chromosome 2. Around 85–90% of these tumors carry the translocation t(2;5) with a fusion of *NPM1* to *ALK* leading to identical *NPM1–ALK* fusion transcripts [39,40,41,42]. In 10–15% of ALK-positive ALCL, *ALK* is fused to one of several other partner genes [43]. The NPM1–ALK fusion protein is expressed in the nucleus and the cytoplasm of the tumor cells, whereas all variant ALK fusion proteins are located in the cytoplasm only. The unique expression pattern of NPM1–ALK allows distinguishing this translocation from other ALK variants by immunohistochemistry staining using the ALK1 antibody. Therefore, minimal disease detection methods targeting the specific *NPM1–ALK* fusion RNA do not require initial fresh tumor material for marker analysis.

Both PCR techniques for *NPM1–ALK* fusion transcripts, as well as the patient-specific DNA breakpoint and flow cytometry, have been explored for minimal disease assessment in children with ALCL. Flow cytometry using surface staining of CD45 and CD30 and intracellular staining of ALK allowed the detection of circulating tumor cells with a sensitivity of 10^−4^ in one early study [44], whereas PCR techniques show a sensitivity of at least 10^−5^ [44,45]. Fusion site-specific PCR-based approaches revealed the presence of circulating tumor cells in blood and BM in 50–60% of NPM1ALK-positive ALCL patients [44,45,46,47,48].

Detection of MDD by the qualitative *NPM1–ALK*-specific reverse transcriptase RT-PCR identifies patients with a significantly higher relapse risk compared to MDD-negative patients in several studies using BFM-type short-pulse chemotherapy [44,45,46,48,49]. The first report of the AIEOP study group detected MDD in BM of 61% of 52 patients at diagnosis. The PFS at 5 years was 41% for MDD-positive patients compared to 100% for patients without detection of MDD (*p* = 0.001) [45]. The prognostic impact of MDD in BM by *NMP1-ALK* RT-PCR was confirmed by the BFM study group in a cohort of 80 patients. The cumulative incidence of relapse of MDD-positive patients was 50% compared to 15% for MDD-negative patients (*p* < 0.001). MDD evaluation in the blood led to comparable results [44]. These data were further validated in combined analyses establishing MDD in blood or BM as an independent prognostic factor, in addition to the histological subtype and ALK-antibody titers [46,48]. In an analysis of the long-term outcome and risk factors among all patients included in the ALCL99 study, only positive MDD measured by the *NPM1-ALK*-specific RT-PCR and the small cell/lymphohistiocytic (SH/LH) morphologic subtype were independently associated with the risk of relapse in multivariate analyses [49].

The possibility to define very high-risk patients by quantifying the *NPM1–ALK* transcripts in BM and blood at diagnosis was analyzed by the BFM study group [44]. Copy numbers detected by a quantitative real-time PCR for *NPM1-ALK* were normalized to the ones of the housekeeping gene *ABL1* (copies *NPM1-ALK*/10^4^ copies *ABL*, NCN). Sixteen patients (21%) with more than 10 NCN *NPM1–ALK* had a cumulative incidence of relapse of 71% compared to 18% for 59 patients with 10 or less NCN in BM (*p* = 0.001). Quantification of *NPM1-ALK* transcripts in parallel blood and BM samples correlated. These findings were recently confirmed in an independent patient cohort of 91 ALCL patients by the same group [50]. Quantitative MDD using the same RQ-PCR-assay in BM and/or blood was evaluated in 60 ALCL patients by the Japanese group. Applying the cut-off of 10 NCN *NPM1–ALK* detected 37% of patients with >10 NCN having a PFS of 58% compared to a PFS of 84% for patients with 10 or less NCN (*p* = 0.0016) [47]. Patients with a very high risk of relapse could not be separated in this study. A comparable finding to the Japanese group was very recently reported by the Children’s Oncology Group with combination therapy of ALCL99 with brentuximab vedotin [51]. These results collectively indicate that copy number quantification using RQ-PCR without stringent quality control between laboratories prohibits comparing normalized transcript numbers from different laboratories.

MDD measurement using flow cytometry using CD30 surface and intracellular ALK staining has only been reported for 11 patients so far. The sensitivity of the quantitative PCR for *NPM1–ALK* outperformed flow cytometry by at least one log [44,52].

A joint analysis of the AIEOP and BFM study group evaluated the prognostic meaning of MRD early during BFM-type chemotherapy by RT-PCR for *NPM1-ALK* [46]. The 5-year EFS of 26 patients with positive MRD before the second course of therapy was 19% compared to 69% for 26 MDD-positive/MRD-negative patients and 85% for 77 MDD-negative patients (*p* < 0.001). The survival for MDD-/MRD-positive patients (65%) was significantly lower compared to MDD-negative and MRD-negative patients (92%). An analysis of early MRD using the same method and MRD-time-point by the French group just confirmed these data [53]. In summary, MRD measurement using a qualitative RT-PCR approach early during chemotherapy allowed the identification of a small patient group (25–30% of all patients) with an 80% risk of relapse and lower survival compared to all other patients [46].

Data on MRD quantification have not been published in a larger cohort so far due to the lack of an internationally harmonized RQ-PCR protocol. However, changes in transcript numbers were successfully used for monitoring the course of individual patients in single laboratories [54,55,56,57].

**Figure 2:**
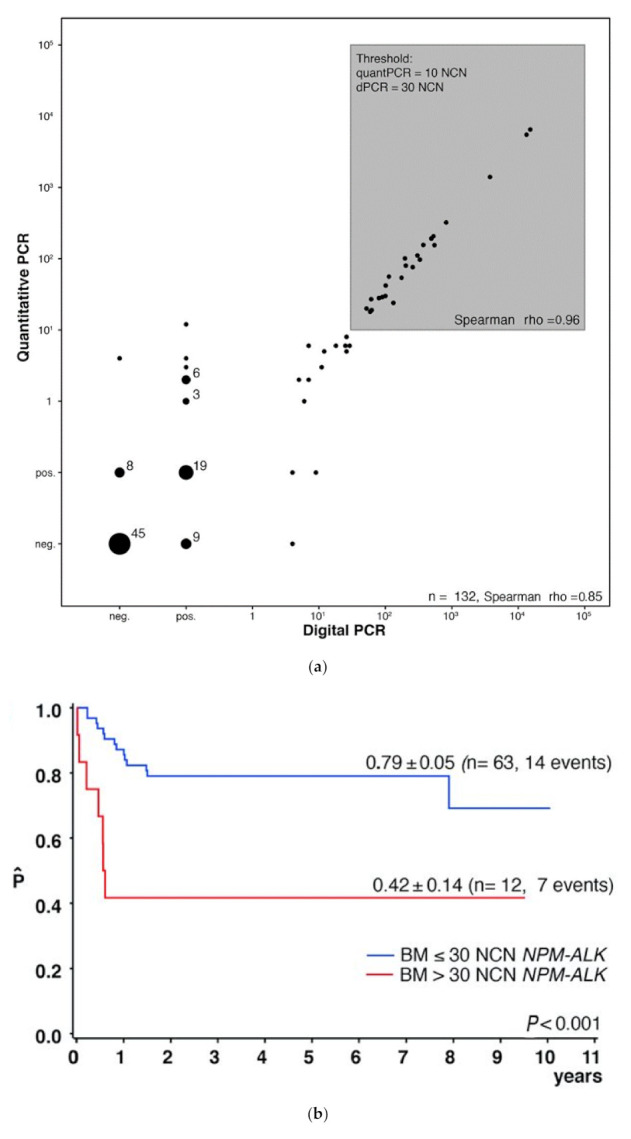
Quantification of minimal disseminated disease by digital PCR for *NPM1-ALK* fusion transcripts in patients with anaplastic large cell lymphoma. (**a**) Correlation of quantification of minimal disease in blood or bone marrow of patients with anaplastic large cell lymphoma measured by RQ-PCR or digital PCR for *NPM1–ALK*. (**b**) Five-year event-free survival (EFS) of 75 children with NPM-ALK-positive anaplastic large cell lymphoma according to minimal disseminated disease in bone marrow quantified by digital PCR using a cut-off of 30 copies NPM1-ALK/104 copies ABL. (from Damm-Welk et al., [50]).

From a clinical point of view, inter-laboratory harmonization of *NPM1–ALK* copy number quantification should have a high priority. Quantification might not only allow for the definition of very high-risk patients initially but could also be used to directly judge the efficacy of new treatment options in very high-risk or relapsed patients, as well as for the detection of impending relapses during follow-up. However, several important obstacles have to be overcome before quantitative measurement can be included in clinical studies. The prognostic cut-off of 10 NCN is near the detection limit of the plasmid calibration curve, and harmonization of the RQ-PCR in quality control rounds, therefore, is challenging, as it was shown for *BCR-ABL* MRD in chronic myeloid leukemia and ALL [58,59,60,61,62]. Quantification of transcripts by digital PCR (dPCR) could be a solution for some of these problems. Digital PCR is independent of calibration curves and can reliably measure especially low copy numbers of target molecules [63,64,65]. The BFM group compared RQ-PCR and dPCR to quantify *NPM1–ALK* transcripts. Copy number estimation by both methods correlated in 132 blood and BM samples (r = 0.85) [50]. Applying a cut-off of 30 NCN *NPM–ALK1* using dPCR allowed the separation of identical patient groups identified by using RQ-PCR with a cut-off of 10 NCN (Figure 2). Quelen and colleagues confirmed the applicability and high concordance of ALK-specific quantification by dPCR compared to RQ-PCR in a series of 49 PB and BM samples from 29 ALK-positive ALCL patients [66]. This indicates that dPCR may allow precise low copy number estimation without the need for standard curves, leading to easier quality control and protocol harmonization in international quality control [50,67].

Compared to the current use of fusion RNA, quantification of circulating tumor cells on the DNA level is not prone to fast degradation and cross-contamination. Investigation of TCR rearrangement as a clonality marker has not been investigated as an MRD target in ALCL so far. Its use is hampered by low tumor cell numbers in the lymphoma and limited tumor material. However, a quantitative dPCR assay using the tumor- and patient-specific *NPM1–ALK* DNA breakpoint was tested for MDD and MRD measurement. The genomic breakpoints are localized in the same introns in *NPM1* and *ALK* [68,69]. Krumbholz and colleagues created a nested multiplex PCR assay to detect the genomic *NPM1-ALK* genomic breakpoints in tumor material. Using the patients’ individual *NPM1-ALK* genomic breakpoints, *NPM1-ALK* genomic DNA in cells and cell-free DNA were quantified [70]. They could show high concordance to RNA-based quantification of fusion gene transcripts. Currently, the major hurdle to a wider application of this method is the necessary fresh tumor material, which is available only from a minority of patients with ALCL. NGS-based methods on FFPE material may become an option to overcome this limitation in the future. Genomic capture high-throughput sequencing was successfully applied for the identification of *V(D)J* rearrangements and genomic translocation breakpoints as MRD markers for precursor B-cell ALL [71]. The usage of genomic DNA extracted from FFPE tumors and ALK-specific capture probes would allow screening for patient-specific genomic ALK translocation breakpoints to establish DNA based MDD/MRD markers without the need for fresh frozen tumors.

Cell-free tumor DNA may be an alternative source to evaluate MRD based on genomic breakpoints. For ALK-positive non small-cell lung cancer, targeted NGS applications were successfully used to identify ALK-specific translocation breakpoints in circulating tumor DNA (ctDNA) [72,73,74]. However, it is not known whether this is transferable to ALK-positive ALCL.

## 5. Data on Minimal Disease in Rarer Childhood NHL

There are currently no systematic data on MDD or MRD in children and adolescents with DLBCL, PMLBCL, or PTCL. The current research on MRD in DLBCL in adult patients uses circulating tumor DNA (ctDNA) and NGS-based applications to detect minimal disease. This was achieved by applying immunoglobulin high-throughput sequencing (IG-HTS) to identify and monitor patient-specific clonotypes or by detecting and monitoring somatic genetic alterations using cancer personalized profiling by deep sequencing (CAPP-seq) [17,18,75]. The analysis of ctDNA of DLBCL patients using IG-HTS or CAPP-seq allowed to associate pre-treatment ctDNA levels with disease stage and tumor burden as well as the outcome or the cell of origin [17,75]. Minimal disease monitoring using ctDNA showed an association of rapid MRD clearance to superior EFS or a longer time to progression [18,76]. CAPP-seq based MRD monitoring during R-CHOP therapy showed rapid disappearance of specific mutated sequences in the cell-free DNA (cfDNA) of responding patients, whereas the detection of mutations persisted in the cfDNA of resistant patients [77].

The feasibility of using TCR rearrangements and flow cytometry for aberrant phenotypes to monitor the course of disease of PTCL was shown in a few studies [78,79]. Tumor clonotypes could be detected in blood and tumor FFPE material by NGS for TCR rearrangements. MRD-positivity at the end of treatment and before autologous blood stem cell transplantation was associated with a high relapse incidence. Taking into account the high relapse rate and rarity of the disease, these data suggest that MRD might be used to guide decision-making for allogeneic blood stem cell transplantation in first remission of PTCL NOS in children [80].

## 6. Future Outlook of Minimal Disease Evaluation in Childhood NHL

From a methodological point of view, the introduction of NGS-based methods for marker screening might solve several current obstacles of minimal disease detection in childhood NHL (Table 1, Table 2a,b). NGS panels could be developed to screen for both genomic breakpoints and mutations as well as IG or TCR rearrangements in both paraffin and fresh tumor material, dependent on the availability in almost all patients [17,18,71]. This would enable the detection of patient or tumor- and patient-specific minimal disease markers on DNA level in most patients. A capture panel for childhood NHL could be developed comparable to ALL [71]. MicroRNAs (miRs) easily traffic among fluid spaces and are more stable than most other RNA classes. Interesting data are emerging regarding their potential role in ALL as MRD biomarkers [81]. The examination of their predictive value for specific subtypes of NHL should be pursued. Furthermore, plasma might be introduced as an MRD medium quantifying ctDNA [13,14,70]. As exemplified for ALK-positive ALCL and other adult lymphomas, dPCR might allow for exact minimal disease quantification even with low copy numbers of the target sequence [50,82].

Given the influence of sample type, sample acquisition, shipment, shipment time, sample preparation, method, and analyses on the result of a minimal disease determination, both pre-analytics, measurements and analyses, need to be harmonized between study groups and laboratories in order to allow using MDD and MRD within international clinical studies [83]. Standardized tissue collection, optimization of methods and media, as well as stringent quality control are to be introduced. These efforts are not easily funded since they are not translational research and not clinical studies but the prerequisite for both. In addition, the different NHL subtypes with low patient numbers necessitate establishing several MRD methods and international collaboration to evaluate the potential of MDD and MRD in childhood NHL.

The potential of MDD and MRD in childhood NHL might go beyond the use as prognostic markers and stratification criteria in clinical studies. Individualized MRD-guided treatment can be envisioned for several subtypes and is close to reality for patients with ALK-positive ALCL. End of treatment MRD might detect high-risk patients for whom a consolidation therapy could be tested to prevent relapse, taking into account the poor outcome in relapse for children with LBL and BL. Whether MRD might be used as a substitute for radiological disease monitoring post-therapy has not been analyzed. Pre-emptive therapy of an impending relapse detected by MRD only could increase the chance of survival of children with a very high risk of relapse. The possible application of MDD at diagnosis of relapse has not been explored either. Given the high predictive value of early MRD in ALCL, MRD might be used as an endpoint in clinical studies testing the efficacy of new drugs or approaches in a window before further standard therapy or for the definition of very high-risk patients eligible for early clinical studies [46].

## 7. Conclusions

The potential of MDD and MRD has just started to be elucidated for children with NHL. The possible prognostic meaning of MDD and MRD using flow cytometry or IG/TCR rearrangements in LBL and using *MYC–IGH* fusion sequences or IG rearrangements in BL/B-AL still needs to be validated. MDD detected by RT-PCR for ALK-fusion genes is an established independent prognostic parameter that serves for stratification in clinical trials. Early positive MRD is ready to be used for the definition of refractoriness in ALCL. Advances and optimization of quality-controlled methods are prerequisites for the quantification of the minimal disease using appropriate markers.

## Figures and Tables

**Table 1:** Minimal disseminated disease (MDD) and minimal residual disease (MRD) in childhood non-Hodgkin lymphoma: possible techniques and available clinical data on lymphoblastic lymphoma (LBL), mature B-cell lymphoma (Burkitt lymphoma/leukemia, diffuse large B-cell lymphoma (DLBCL), primary mediastinal large B-cell lymphoma (PMLBCL)), and peripheral T-cell lymphoma (PTCL).

	Lymphoblastic Lymphoma		Burkitt Lymphoma		DLBCL, PMLBCL	PTCL
Marker	IG/TCR Rearrangement	Aberrant Marker Expression	IG Rearrangement	MYC–IGH Fusion Site (DNA)	IG Rearrangement	TCR Rearrangement
Techniques	Marker screening: PCR, NGS Quantification: RQ-PCR	Flow cytometry	Marker screening: PCR, NGS Quantification: RQ-PCR	Long-distance PCR	Marker screening: PCR, NGS Quantification: RQ-PCR	Marker screening: PCR, NGS Quantification: RQ-PCR
Applicability	Most patients with initial tumor	Most patients	Most patients with initial tumor	65–70%	?	?
Sensitivity	10^−5^	10^−4^	10^−4^	10^−3^–10^−4^	10^−4^–10^−5^	10^−5^
Initial Tumor Material	needed	not necessarily	needed	needed	needed in most cases	needed
Clinical data on MDD	-	+	+	+	-	(+)
Clinical data on MRD	-	(+)	(+)	+	-	(+)

-, no clinical data; + one study demonstrating a prognostic value; (+) prognostic value possible; data not clear; ? not known.

**Table 2:** Minimal disease detection for children with anaplastic large cell lymphoma (ALCL).

(**A**) Methods to measure minimal disease in ALK-positive anaplastic large cell lymphoma (ALCL).				
	**RT-PCR NPM–ALK Transcripts**	**RQ-PCR NPM–ALK** **Transcripts**	**dPCR NPM–ALK** **Transcripts**	**RQ-PCR** **for DNA Break**
Applicability	85%	85%	85%	n.k.
Sensitivity	≤10^−5^	≤10^−5^	≤10^−5^	≤10^−5^
Advantage	easy QC, inexpensive	allows following response to therapies	High sensitivity QC easier compared to RQ-PCR	Patient-specific ctDNA detectable
Disadvantage	no quantitative response monitoring	difficult to harmonize, expensive	expensive	fresh tumor needed, expensive, laborious
(**B**) Established clinical applications for minimal disseminated (MDD) and minimal residual disease (MRD) in ALK-positive anaplastic large cell lymphoma (ALCL).				
**Specific Marker**	**MDD/MRD**	**Patients** **Positive (%)**	**Clinical** **Relevance**	**Specific Marker**
RT-PCR for NPM–ALK Transcripts (RNA)	MDD MRD	50–60 25	HR patients (50% EFS), validated [44,45,46,48,50] VHR patients (25% EFS), validated [46,53]	RT-PCR for NPM–ALK transcripts (RNA)
RQ-PCR or dPCR for NPM–ALK Transcripts (RNA)	MDD MRD	20–25	VHR patients (30% EFS) [44,50] Individual response to therapy [54,55,56,57,70]	RQ-PCR or dPCR for NPM–ALK transcripts (RNA)

n.k. not known; ctDNA, circulating tumor DN;, QC, quality control; HR, high relapse risk; VHR, very high relapse risk.
